# P-442. Quantification of TTV in Saliva and Plasma of People Living With HIV

**DOI:** 10.1093/ofid/ofae631.642

**Published:** 2025-01-29

**Authors:** Edmund Gore, Hester Groenewegen, Annechien Lambeck, Bert Niesters, Wouter Bierman, Coretta Van Leer Buter

**Affiliations:** University of Groningen, University of Groningen Medical Centre, Groningen, Groningen, Netherlands; University of Groningen, University of Groningen Medical Center, Groningen, Groningen, Netherlands; University of Groningen, University of Groningen Medical Centre, Groningen, Groningen, Netherlands; University Medical Center Groningen, Groningen, Groningen, Netherlands; University of Groningen, University of Groningen Medical Centre, Groningen, Groningen, Netherlands; University Medical Center Groningen, Groningen, Groningen, Netherlands

## Abstract

**Background:**

Torque tenovirus (TTV) viral load in blood is now accepted as a marker of immunocompetence in immunocompromised patients such as transplantation recipients and people living with HIV (PLWH). TTV has been shown to predominantly infect T cells, which are vital for the control of TTV. In PLWH the level of TTV has been shown to correlate with the CD4 count. Recently, TTV has also been detected in saliva of PLWH. However, at this time it is not known if salivary TTV can be used as a non-invasive alternative to blood TTV, or as an alternative to a CD4 count. In this study, we explore the relationship between TTV in blood and in saliva with T cell subsets, including CD4^+^ T cells, to determine the clinical potential for this novel marker in PLWH.

**Figure:**
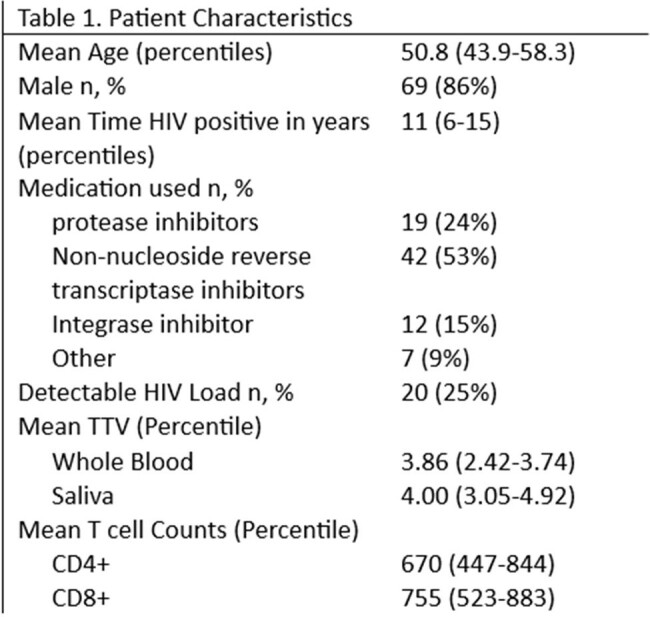

**Methods:**

80 PLWH visiting the outpatient clinic of our center were recruited. Inclusion criteria were age ≥ 18 years, use of combination antiretroviral therapy for ≥ 6 months and < 40 copies/mL in the last two viral load measurements. The CE-marked TTV R-Gene® kit (bioMérieux, Lyon, France) was used to detect and quantify TTV in plasma and saliva. Results are expressed in log copies/ml, with a cut off of 2.4 log copies. Absolute numbers of CD4+ T cells were measured using the MultiTest TruCount method (Becton Dickinson). HIV viral load was performed on EDTA plasma samples using the Abbott Real-Time HIV-1 assay.

**Figure:**
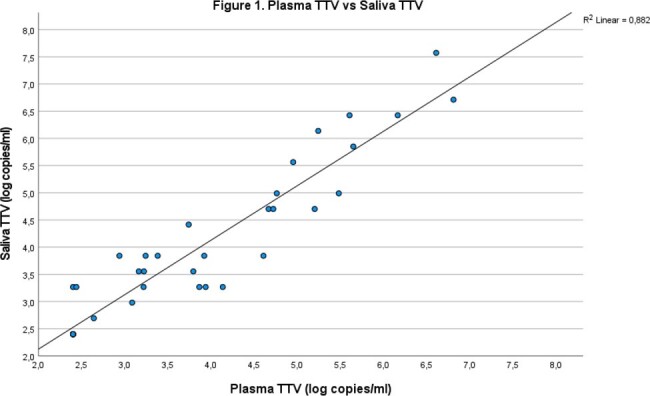

Plasma TTV correlates with salivary TTV, R2 0.89, p<0.01.

**Results:**

Individuals were predominantly male (86%), had a mean age of 51. The mean CD4+ count was 670 (447-844) and 25% had a low detectable HIV load (Table 1). TTV was detectable in 24% of plasma and 20% of saliva samples with an average of 3.86 log copies/ml and 4.01 log copies/ml respectively. Plasma TTV and saliva TTV correlate well (R^2^ 0.89, p< 0.01) (figure 1). There is no relationship between plasma TTV (R^2^ 0.004, p = 0.58), (figure 2) or saliva TTV and the CD4+ count (R^2^ 0.004, p = 0.58) (figure 3).

**Figure:**
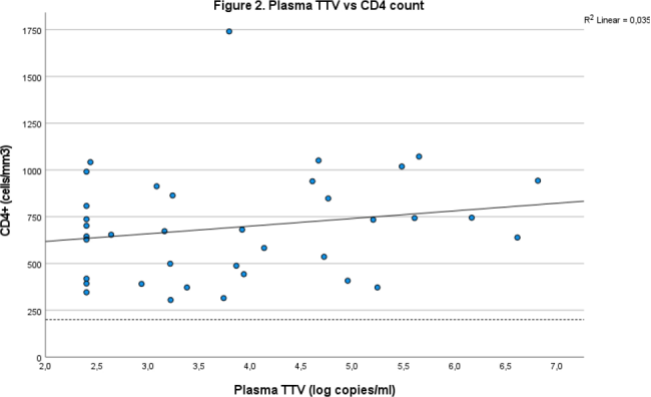

Plasma TTV does not correlate with CD4 count, R2 0.004, p = 0.58.

**Conclusion:**

Whilst neither salivary nor plasma levels of TTV correlate with the CD4 count of HIV patients in our study, salivary TTV correlates with TTV detected in blood. With the increasing interest in TTV in transplantation as a marker for immunosuppression, the fact that measurements of TTV can be performed on the saliva, preventing or reducing the need to draw blood from patients deserves further exploration.

**Figure:**
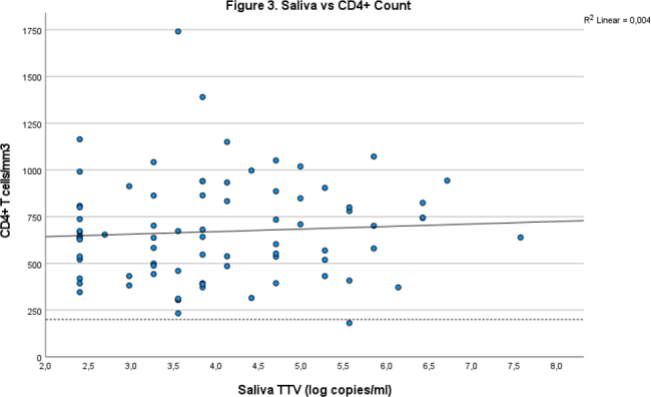

Salivary TTV does not correlate with CD4 count, R2 0.004, p = 0.58.

**Disclosures:**

**All Authors**: No reported disclosures

